# Considering “the more” of patients suffering from alcohol use disorders. An illustration of acute nursing care from a lifeworld-led perspective

**DOI:** 10.1080/17482631.2020.1783860

**Published:** 2020-06-30

**Authors:** H. M. Bové, M. Lisby, N. Brünés, A. Norlyk

**Affiliations:** a Research Center for Emergency Medicine, Aarhus University Hospital, Aarhus, Denmark; b Section for Nursing, Department of Public Health, Aarhus University, Aarhus, Denmark; c Amager og Hvidovre Hospital, Denmark

**Keywords:** Acute nursing care, nursing care, alcohol use disorders, substance abuse, qualitative research, lifeworld-led care, nursing practice

## Abstract

**Purpose:**

The purpose of this study is to illustrate a theoretical value framework for humanisation of healthcare, a lifeworld-led care that has the potential to support nurses in acute medical units in addressing and meeting both challenges and care needs expressed by patients suffering from alcohol use disorders. Providing care to these patients means working with a very divergent and complex group of patients. When hospitalised in an acute medical unit, nurses are often these patients' first encounter, which gives a unique opportunity to initiate and establish a successful care alliance.

**Method:**

The present study is a qualitative study based on an amplified secondary analysis of 25 pre-conducted interviews. Following a hermeneutic approach, the analysis was structured in accordance with the conceptual value framework for humanisation of care, drawing on the recognition of the patients' lifeworld as an aspect of importance.

**Findings:**

The study showed that while there were examples of humanising care guided by the patients’ lifeworld present, there were also situations of care that were dehumanising. **Conclusion:** When letting the patients’ perspective of well-being be the centre of care, the patients’ experience of meaningfulness and sincerity within the provided care was nurtured, and they felt more humanly met.

## Introduction

In 2007, the Danish Health Authority recommended a reorganization of the already existing emergency departments. Acute medical units (AMU) were established to promote a more efficient emergency admission process and to improve the quality of the general medical care and treatment (Sundhedsstyrelsen [The Danish Health Authority], 2007).

The primary focus in the AMUs is to assure timely and correct diagnoses and the initiation of relevant treatment as soon as possible. All incoming patients are triaged in accordance with their need for treatment and care. The triage system is based on lean principles of patient flow, the main objective being to detect the seriously ill patient and to provide guidance for healthcare personnel (Nordberg et al., 2010). Furthermore, an early warning score and an alcohol-withdrawal score are applied, the latter only for patients presenting problems related to excessive alcohol consumption. The treatment for patients considered to be suffering from an excessive consumption of alcohol often involves alcohol detoxification, medical prevention of alcohol-withdrawal symptoms, and if relevant treatment of other clinical conditions related to the admission. An aim of the acute nursing care and treatment is to assess the patient’s physical and bio-medical needs, transfer patients to higher dependency units for further treatment, or to discharge them. Consequently, the acute nursing care is high paced, time-dependent, and based on short-term treatment.

Patients suffering from alcohol use disorders (AUD) are a particularly vulnerable and complex group of patients (Bartlett & Brown, 2013; Becker & Semrow, 2006). When these patients are admitted to hospital, they are placed in an additional, vulnerable position. Several studies have shown that these patients experience stigmatization, unjust behaviour, anger, etc., probably deriving from preconceived and unfounded opinions among health professionals (Link & Phelan, 2006; Lovi & Barr, 2009; Schomerus et al., 2011; Williamson, 2012; Yang et al., 2007). This unveils as patient-experienced reservations in the relationships with nurses (Indig et al., 2009; Neville & Roan, 2014; Talbot et al., 2015), which may affect the care provided during a hospital stay. Moreover, studies have clarified that these patients may be considered responsible for their own misfortune (Järvinen, 1998; Kvamme et al., 2015; Lovi & Barr, 2009; Schomerus et al., 2011; Williamson, 2012), thus demonstrating that prejudice and negative stereotypical views often cause barriers to treatment by reducing the nurses’ general willingness to provide care (Chung et al., 2003; Indig et al., 2009; Skinner et al., 2009). Hence, providing care for this group of patients can be a complicated and difficult task (Bové et al., 2019; Roche & Pidd, 2010).

Recent research elucidates that healthcare professions need to be aware of the need for mental health and psychosocial support to patients suffering from AUD who repeatedly attend emergency departments (Parkman et al., 2017). Also, research has emphasized that this patient group calls for an authentic presence characterized by an intentional attentiveness from the nurses to help ease the acute medical hospitalization (Bové et al., 2018). Thus, the above studies indicate that within the current focus and organization of care in the AMUs, an increased attention towards the individual patient’s perspective is essential. However, integrating patients’ perspectives into the care requires an open attitude towards the individual patient’s lifeworld in order to engage in the patient’s subjective perspective of health and well-being (Benner & Wrubel, 1989; Dahlberg et al., 2009; Martinsen, 2010; Todres et al., 2007; Travelbee, 2010). Galvin & Todres (2013) bring forward a perspective of care that focuses on upholding a particular humanizing view on what it means to be human. The value framework of lifeworld-led care addresses the vulnerability of human existence and points out a direction for a sensitive practice of care that emphasizes the individual’s lifeworld and experience of well-being and does not solely focus on the absence of illness; “*[…] something less measurable but keenly felt”* (p. 1). In an acute caring context, this framework seems highly relevant as research highlights that both patients and healthcare professionals are requesting increased attention to the uniqueness of the individual (Bové et al., 2018, 2019).

Based on the above humanized value framework of Galvin and Todres (2013) the purpose of the present study is to illustrate how caring practice, inspired by this framework of lifeworld-led care has the potential to support nurses in acute medical units in addressing and meeting both challenges and care needs expressed by patients suffering from AUD.

## Design and methodology

### Theoretical framework

The theoretical value framework of lifeworld-led care builds on the tradition of the continental philosophy of Husserl, Heidegger, and Merleau-Ponty (Dahlberg et al., 2009; Galvin & Todres, 2013). The fundamental idea of the humanization of care is based on Husserl (1936/1970) thinking of humans existing through their personal perceived world, the lifeworld. Husserl described the lifeworld as the ongoing flow of experiential life in which the world is experienced and lived (German erlebt): “*everyday surrounding world of life […] we are objects among objects in the sense of the lifeworld, namely, as being here and there”* (Husserl, 1970, pp. 104–105). The lifeworld thus describes a person’s subjectively experienced world that becomes valid through an inter-subjective consciousness of fellow humans and existing through “a living together-ness”. Heidegger, 2002 further developed this thinking and elaborated on how technological progress might increase the distance between the subjective experience and the objectively lived world. The framework for humanizing of care thus provides a comprehensive value base of how to retain care, emphasizing the closeness between the subjective experience and the objectively lived world (Galvin & Todres, 2013). This involves eight dimensions of humanization (Table I) to be considered in both caring systems and caring interactions; more specifically to ask the question: what needs to be the centre of the nurse’s attention to make the patients feel more deeply met as humans? (Galvin & Todres, 2013).

**Table I.** Eight dimensions of humanization (Galvin & Todres, 2013, p. 11).

Forms of humanization	Forms of dehumanization
Insiderness	Objectification
Agency	Passivity
Uniqueness	Homogenization
Togetherness	Isolation
Sense-making	Loss of meaning
Personal journey	Loss of personal journey
Sense of place	Dislocation
Embodiment	Reductionist body

The following eight dimensions are not separate absolutes or unconnected concepts. Instead, the dimensions imply one another and are thus interrelated and intertwined. To get a comprehensive understanding of what humanization of care means, the focus of care needs to point towards a lifeworld-led approach to care that emphasizes the existential view from the inside of the patient (Galvin & Todres, 2013).

## Method

To illustrate how the framework of lifeworld-led care can support nurses in addressing and meeting challenges and care needs as expressed by both patients suffering from AUD and nurses caring for the patient group. The present paper thus draws on data from two previously conducted empirical studies that elucidate patients’ and nurses’ experiences of collaborating and engaging in relationships as care receivers and care providers. An amplified secondary analysis (Heaton, 2008) of the original data was performed to address new questions and perspectives based on the eight dimensions of humanization and/or de-humanization (Galvin & Todres, 2013). The amplified secondary analysis thus allowed for revisiting and combining the original data material into new ways by addressing new questions that subsequently enabled an analytic expansion of the original data.

### Context of the two studies

The original empirical data were collected in a 53-bed AMU at a university hospital in Denmark by the first author. The AMU was divided into three minor sections each with individual head nurse in charge.

The data consisted of 15 in-depth phenomenological interviews with patients suffering from AUD and/or alcohol dependency admitted to one of the three units (Bové et al., 2018) (study I) and 10 in-depth phenomenological interviews with nurses providing care to this patient group in the same AMU (Bové et al., 2019) (study II). All 25 interviews concerned participants’ experiences related to care within the AMUs. All interviews were recorded and subsequently verbatim transcribed.

In study I participants were identified on the unit’s electronic admission boards by the first author, during a 4-month period in 2016. Eight males and six females participated. They were aged between 32 and 78. One participant withdrew from the study due to logistic reasons.

The participants were interviewed once towards the end of their hospitalization. The interviewer had no former relationships with the participants.

While the participants were hospitalized, they were triaged as part of the medical procedures by the Danish Emergency Process Triage to determine treatment urgency (Nordberg et al., 2010). An early warning score and an alcohol-withdrawal score were likewise measured regularly in accordance with both local recommendations and the patient’s clinical condition. Reasons for admission differed among the participants; however, they were all treated medically with benzodiazepines while hospitalized.

To recruit participants in study II information about the project was distributed both virtually to the group of clinical nurses in the unit and via leaflets in the staff room(s). We included nurses who were particularly knowledgeable about the study phenomenon, i.e., providing care to patients suffering from AUD, to obtain rich data (Dahlberg et al., 2008). Further, in order to gain a deep understanding of the phenomenon the participants were included with an attention towards participant variation (Norlyk & Harder, 2010). The nurses represented a wide range of nursing experience, their job duration was between one and 23 years, the nurses were aged between 26 and 57 years, and one male and nine females were interviewed. They were interviewed once. The interviews were conducted in an adjacent room and nursing tasks were reassigned to colleagues in order to reduce interruptions during interviews.

Below, the findings of the two studies are summarized in Table II.

**Table II.** Overview of studies I and II.

	Study I	Study II
Title	Scheduled care—as a way of caring. A phenomenological study of being cared for when suffering from alcohol use disorders (Bové et al., 2018)	Do the carers care? A phenomenological study of providing care to patients suffering from alcohol use disorders (Bové et al., 2019)
Aim	To elucidate the lived experience of how patients suffering from AUD experience being cared for when admitted to AMUs	To elucidate nurses’ lived experience of providing care to patients suffering from AUD.
Method	Phenomenological studies using Reflective Lifeworld Research (Dahlberg et al., 2008).	
Findings	Being cared for when hospitalized was experienced as a two‐stage process. *Stage 1*: “Scheduled care experienced as caring” was related to the first part of the hospitalization	Providing care to patients suffering from AUD was a highly challenging and demanding task for nurses working in the AMUs. A need to engage with the patients in a sensitive collaboration was identified concerning the nurses’ ability and willingness to be open and to adjust the care accordingly.
	Patients’ centre of attention was on avoiding alcohol-withdrawal symptoms. An experience of an authentic presence provided by the nurses made the patients feel acknowledge in both agency and vulnerability.	Within the sensitive collaboration, a two-sided feeling of responsibility was revealed.
	*Constituents* being in a safe haven sharing a tacit but mutual goal	A professional responsibility related to a task-oriented approach to caring A personal responsibility and obligation related to adopting a caring approach focusing on being with the patient.
	*Stage 2*: “Scheduled care experienced as non‐caring” was related to the second part of the hospitalization and gradually set in as the blood alcohol level decreased and trust in the nurses increased. A transfer of attention within the participant was identified. *Constituents* being in a chaotic space being on your own	*Constituents* Dealing with the intricacy of the patient’s life situation Balancing care between standardized procedures and a complex patient Being caught between responsibility and resignation
	Being met in an authentic presence made a decisive difference to the patients and encompassed the experience of enabling a positive trusting collaboration to emerge.	
		The discrepancy between the two approaches created feelings of despondency and resignation, hence an inability to provide the care the nurses wished to give.

### Ethical considerations

To avoid the participants of study I to feel coerced contributing to the study, they were approached by the nurse caring for them and asked if they would accept meeting with and possibly be interviewed by the first author. The present study was completed in accordance with the ethical considerations and principles of conducting medical research set out in the Helsinki Declaration (World Medical Association, 2013) All participants were given oral and written information about the study, and verbal consent was achieved before the interview. Participants were guaranteed confidentiality and signed a written consent form before the interviews. Further, the study was approved by the Danish Data Protection Agency (1–16–02–90–16).

### Data analysis

The theory-driven approach to humanization of care was used as a conceptual schema to structure the analysis of the present study. No software was used to analyse the data; instead, the analysis and its findings were elaborated intensively with the group of authors who remained open, reflective, and sensitive to nuances and changes in meaning in the material. The analysis thus materialized as an iterative process of going back and forth between theory and interpreting data through which a new understanding was developed. Hence, the analysis followed a classical hermeneutic circle by working in a spiral movement between the whole and smaller parts of the text (Gadamer, 1995). As a first analytical step, all text passages from the 25 interviews that gave answers to the enunciated analytical questions: “*What does it take to make patients suffering from AUD feel humanly met?”* and “*When do these patients feel humanly met?”* Secondly, all selected text passages were read, re-read, and structured on the basis of the eight dimensions of humanization (Table I).

## Findings

The findings are structured according to the eight dimensions of humanization (Galvin & Todres, 2013). Quotations from the two previous studies are provided as examples to illustrate the particular points.

### Insiderness/objectification

Insiderness is related to the “inside-world” of people. Only people themselves can know with certainty what this inside sense is. It is this subjectivity that gives people the sense of feeling, mood, and emotion that gives the lifeworld meaning and value (Galvin & Todres, 2013). In study I, the patients experienced that the nurses were attentive and available during the first part of the hospitalization, creating a feeling of being in a safe place and making the hospitalization more manageable. The patients felt met as human beings, with an individual personality and in a mutual understanding of not being alone:

*They had already taken notice of me and arranged something suiting me before I’deven entered the unit’*.

The awareness and attentiveness towards the patients’ being created in the patients a feeling of being safe and cared for; thereby illustrating insiderness as an individual inside sense emphasized by the nurses in the care.

Objectification is seen when people primarily focus on how people fit into a diagnostic system, i.e., a standardized procedure that does not consider the individuality of the person involved (Galvin & Todres, 2013). In study I, the patients described that the experience of the organizational structures and procedures changed during the last part of the hospitalization because the care during this part of the hospitalization did not deviate from the stringency demanded by the procedures. A feeling of objectification and not being seen as a fellow human occurred. Objectification was described by the patients as having their personhood diminished and being viewed as a patient that needed to fit into the system demanded by the procedures:

*The feeling of not being understood was appalling*.

Similarly, nurses in study II described how they at times felt coerced to make the patients fit into a predefined mould:

*They [patients] need to fit in […]and then we can say we did it […]*.

Being able to meet and see patients in both insiderness and objectification may balance the care in a way that enhances a feeling of being a fellow human in patients suffering from AUD.

### Agency/passivity

Being responsible and making personal decisions is an essential part of agency. Freedom to be and do creates a sense of agency in which we are formed as human beings (Galvin & Todres, 2013). In study I, patients’ experiences of sharing a tacit but mutual goal with the nurses empowered senses of agency:

*I don’t hesitate, I just ring the bell. I don’t ask, I just tell them [nurses]*.

Feeling involved and being given the possibility of freedom in terms of acknowledging the patients’ way of being and acting promoted a sense of agency. The sense of agency had a positive impact on patients’ experiences of the hospitalization because it created the feeling that regaining a sense of self was achievable.

The dimension of passivity has to do with the attitudes and practices that render a person passive in relation to their condition (Galvin & Todres, 2013). In both study I and study II, passivity occurred as a multifaceted concept. Nurses described difficulties in comprehending passivity and non-engagement of the patients:

*[…] they [patients] are just there.[…]they are just [emphasises “just”] lying there[…]*.

The experience of patients’ passivity appeared when neither involvement nor participating was present, i.e., when diagnostic procedures and/or nursing tasks took place without any engagement of the patient. Furthermore, work routines and hospital structures seemed to render the patients’ passive by not encouraging participation because participation only happened if the nurses induced it:

*[…]I need to be sure he can handle it [self-administration of medicine] […] andshow he can keep an appointment […]*.

Nevertheless, patients in study I described how they at times deliberately chose to be passive:

*[…] just let me be. Leave me alone I just want to be here, lie right here […]—I don’tcare*.

This dimension of passivity illustrates the complexity of providing care to this group of patients because despite the humanizing sense of agency, there may be a necessary passivity related to caring. For patients suffering from AUD, acknowledging the passivity requires adjustment, reflection, and knowledge in order to warrant patient passivity as a sense of agency and thus a way of feeling a freedom to be and do.

### Uniqueness/homogenization

Perceiving the uniqueness of a person is to acknowledge that human beings are more than the sum of their parts. Study I elucidated that even small nuances in the care were noticed and made the patients feel acknowledged and seen as fellow individuals:

*[…] I get the feeling that they [nurses]see me. […] I notice and it matters*.

By taking the subjective perspective of the patients into consideration, the provided care strengthened the feeling of uniqueness within the patients.

Homogenization centres on decentralizing the uniqueness of humans by making them fit into a particular group. Homogenization appeared in both studies when the nurses objectified the patient in a way in which the scheduled care became so principal that the patient experienced being decentralized as a person:

*I’m so much more than this […]*.

Accordingly, when the provided care became procedural and equivalent to actions and decisions predicted by an algorithm, this rendered the nurses into not seeing the particular of the patient:

*The most important thing is to find out how the patient is doing, and to do that I havethe early warning score […]*.

The feeling of being unmet as a human was expressed as:

*They [nurses] know everything about withdrawal symptoms, but nothing aboutalcoholism […]*.

Thus, to become aware of and keep the uniqueness of the patient suffering from AUD means to be able to or have the courage to adjust the care accordingly.

### Togetherness/isolation

Togetherness exists as a grounded content sense of being a part of something and thus emerges in relation to others. Togetherness was identified when patients experienced the feeling of being part of a team, i.e., collaborating on alleviating the suffering and thus being empowered to becoming better:

*[…] we are in this together. I’m not alone—fighting*.

Likewise, some patients seemed to reject togetherness and chose to live in a self-imposed aloneness. However, the feeling of empathy and belonging was essential, enabling the patients to better comprehend suffering:

*[…] If I had two days left and one had to be a sick day but the second day wouldimply a shower, then I’d easily manage day one*.

The nurses in study II emphasized that alleviating the patient’s suffering also concerned an attentiveness and supporting the patient going through a difficult time:

*I can’t fight for them, though I can fight with them […]*.

Isolation exemplifies itself when people find themselves detached from being a part of something, not having a sense of belonging together in solidarity (Galvin & Todres, 2013). The feeling of being alone and disconnected from togetherness also appeared in study I, making the patents suffering from AUD feel despondent and isolated:

*You are just all alone in here just deal with it […]*.

However, the nurses in study II did demonstrate that a distinct attention towards the feelings of isolation and a consideration for how to provide a meaningful and individual care were present:

*The dilemma of the day is always, do I want to stick with the procedures and followthe rules, or do I want to provide a decent and more suitable care*.

Accordingly, feelings of togetherness emerge when the patient suffering from AUD is implied in the care alliance in a way that allows for both connectedness and aloneness to be a sense of mutual belonging.

### Sense-making/loss of meaning

Sense-making is a feeling or motivation of bringing things together that creates meaning. Patients in study I experienced sense-making when they were involved in the care and treatment, thereby bringing limpidity to the care alliance:

*It [information] matters and now I better understand […]*.

Nurses in study II likewise experienced sense-making when they engaged with the patients in a sensitive cooperation, thus grasping what really mattered:

*We always try to do our best for things to actually happen […]things that meansomething*.

The caring approach of considering and meeting the patients suffering from AUD in a sensitive cooperation thus created a mutual feeling of meaning and integrity for both of them within the care.

The opposite of sense-making is loss of meaning characterized by a feeling of not being considered, but instead just counted as a statistic (Galvin & Todres, 2013). The loss of meaning appeared in study I when patients experienced a feeling of being alienated due to standardized procedures (Bové et al., 2018). Likewise, the nurses in study II experienced loss of meaning when the feeling of being unsure if the provided care was right and helpful to the patient:

*[…] when you realise one of them is on their way in here again, you kind of just giveup—before they are even here […]*.

Patients suffering from AUD may experience a loss of meaning when sense-making is obscured and feelings of not being considered but instead being a number in a “tick-off” or “to do-list”.

### Personal journey/loss of personal journey

Personal journey unfolds around the life experienced as a (meaningful) excursion through time. Knowledge about temporality as a dimension to the lifeworld is therefore necessary in order to bring patients’ perspectives into the case history, letting these perspectives lead the caring practice. The nurses in study II described how implying the patients’ stories within the care became a tacit knowledge to both ease and provide care:

*You kind of get to know them [patients] you talk to them and find out who they are[…] everything is easier then […]*.

Including the patients’ stories into the care led to a facilitating collaboration. Nurses experienced that by being familiar with the patients’ past, their personal journey was better supported during the caring encounters.

However, according to the patients’ experiences in study I, a loss of a sense of being on a personal journey became present when they experienced their narrative becoming lost in the caring practice:

*I’m not sure if they really take me into consideration—only what needs doing […]They [nurses] know everything about withdrawal symptoms, but nothing aboutalcoholism […]*.

In order to support patients suffering from AUD positively towards the future, nurses need to be attentive to achieving a sense of the patient’s personal continuity, hence to look beyond the present and engage with the temporal dimension of the patient’s lifeworld and thus create an experience of meaningfulness within the caring practice.

### Sense of place/dislocation

Everybody comes from somewhere, a particular place where a feeling of at-homeness is essential (Galvin & Todres, 2013). A sense of place was identified when the patients’ feeling of at-homeness was supported during the hospitalization. This was characterized by a feeling of locality and being settled, despite the power of being on the inevitable treatment-pathway:

*[…]it is a nice feeling that there is someone there […]that someone still has anopinion about what I should be able to do or not do […] nobody has an opinion aboutthat […]*.

Dislocation occurred as a feeling of being left back in nothingness in a meaningless space with no attention being paid to either the quality of space or how to support the patients’ at-homeness. In study I, a state of self-marginalization and aloneness emerged which challenged the patients in adjusting to the new culture and created a feeling of being or becoming a stranger:

It’s not easy being here[…].

Nurses in study II were aware of this:

*You can’t blame them really […]imagine going through hell not knowing where,what, when, and why*.

Hence, feelings of at-homeness and of being a part of something were essential to patients suffering from AUD when hospitalized in an alien environment. It is thus important that the nurses recognize the influence of spatial quality on patients’ feelings of meaningfulness and well-being.

### Embodiment/reductionist body

Embodiment is a form of idea or feeling closely associated with our insiderness, i.e., bodily grounded messages like pain and hunger or inside messages related to a being-in-the-world as senses of meaning or vitality (Galvin & Todres, 2013), a personal experience of one’s own body. In study II, nurses found it challenging to fully grasp and imagine what the patients’ lives were like, and this was illustrated by a caring practice that separated the patient from their individual subjectivity and meaningfulness:

*[…] he keeps coming here with the same abuse issues to sort out every single time,and you just don’t get it*.

Patients described how a reductionist view of the body was experienced to be dominant in the medical context:

*It’s not about me, it’s about my body […]*.

This exemplified that anything other than a view of the body as a personal experience is a reductionist view of the body. It was obvious how focus of care relied mainly on signs and symptoms and on the physical body as an object to fix:

*They [nurses] know everything about withdrawal symptoms, but nothing aboutalcoholism […]*.

Nurses providing care to patients suffering from AUD may have to consider the patients’ unique experiences of their bodily expressions in order to becoming able to respond to the patients’ bodily expressions in a meaningful way and thus not provide a reductionist care.

## Discussion

The findings in the present study highlight examples of both humanizing and dehumanizing dimensions of care exemplified in an acute caring context. The findings elucidate that by letting the individual patient’s perspective of well-being be the centre of care, an experience of meaningfulness and sincerity within the provided care is nurtured. When nurses engage in the patients’ perception of their lifeworld and well-being by, for example, not letting the predefined actions stipulate the care, a strengthening of patients’ insiderness and uniqueness is created. Hence, promoting feelings within the nurses of not solely providing a standardized care but an individual care directed by the subjectivity of the patient. The findings further elaborated that the organization of care within the AMUs framed the nursing practice in a way that inevitably positioned the caring practice in a schism of either following the standardized procedures or deviating and shifting towards a more individual and sensitive care. The standardized and procedural way of organizing care has previous been elucidated in order to draw attention to whether the practice of care should be technically or judgement based (Polkinhorne, 2004). Nevertheless, research has explored the continuum of care and cure, thereby elaborating that the biomedical focus, aiming at diagnosis and potentially cures, has an underlying presumption of care, because the non-physical aspects of illness are less valued. This creates a dichotomy between care and cure within the caring practice (Borrell-Carrió et al., 2004; Nyström, 2002; Russell, 2014; Treiber & Jones, 2015). Following this, Ernst (2016) found that manoeuvring in-between the dichotomy of care and cure takes a more flexible and pragmatic approach to “best practice” of nursing. Accordingly, the findings in our study point out that while the mindset of cure may be recognized as generating better medical results and improving patients’ safety, ignoring the caring dimension may take nurse’s experience of meaningfulness and enthusiasm out of practice. This can cause complex situations where the standards cannot adequately direct appropriate action and may render practice more unsafe.

In our findings, the dichotomy between care and cure appeared in most of the dimensions, although the dichotomy was particularly apparent within the dimension of uniqueness/homogenization. Our findings show that caring for patients suffering from AUD also involves a state of being with the patient. However, balancing care in-between doing and being was challenging, and showing the determination of being in the atmosphere of doing in the AMUs was rarely achieved. Kirk and Nilsen (2015) found that the atmosphere and culture within a Danish emergency department are highly characterized by activity, acts of doing and performing in the clinic. Nevertheless, the findings in the present study illustrate that when an imbalance between doing and being appeared, patients experienced their uniqueness de-emphasized.

Our findings also elaborate on the importance of understanding the patient in the context of the personal journey. Meaning that when nurses are inattentive to the context of the patient, the experience of meaningfulness is at risk of becoming lost. The personal journeys of both patients and nurses relate closely to the concept of temporality which brings humans into history. To engage in a caring perspective with an understanding of temporality and support of the personal journey of the patient suffering from AUD means involving a nuanced perspective of being with the patient. In other words, taking the patients’ perspectives and their way of being in the world into account (Dahlberg et al., 2008; Galvin & Todres, 2013). However, temporality can also be interpreted as the linear progression of time (Caldas & Berterö, 2012; Dahlberg et al., 2008). Our findings elucidate the necessity to understand both the engagement with and the complexity of temporality.

It was also shown in the findings that patients experienced feelings of being alone and isolation during the hospitalization. Andersson et al. (2012) found that care activities in an emergency department may be performed in a more mechanical than in a caring way due to the standard medical management and time pressures. According to Galvin and Todres (2013), the feeling of isolation may become present when procedures and standardization take away the sense of belonging. In our study, these existential feelings of aloneness were identified when the provided care became procedural and equivalent to actions predicted by the algorithm of the procedures, thus rendering the nurses not to see the particularity of the patient. The present study thus draws attention back to the fundamentals of nursing in terms of elucidating that there still is more to care than cure, which is much needed according to previous research within the area of patients suffering from AUD (Lovi & Barr, 2009; Neville & Roan, 2014).

The provision of acute nursing care in the AMUs may necessarily encompass a certain degree of procedural and systematized caring, nevertheless our findings illustrate the importance of nurses adopting an approach of caring, emphasizing, and focusing on a being with the patient, thus humanizing the systematized and scheduled care through an attentive and sensitive cooperation. Our findings show that the sense of belonging and togetherness may be vulnerable in an in-hospital context. Moreover, the nurses’ attention needs to be on both the patients’ experience of being cared for and the nurses’ own experience of providing a suitable and meaningful care. The findings also illustrated that patients’ feelings of well-being could be enhanced due to the experience of togetherness in relation to the nurses. It became apparent how togetherness created a mutual understanding of the patient’s suffering, which created a strong care alliance and enabled the patient suffering from AUD to better comprehend the suffering. Revisiting the fundamental values of nursing care, research on suffering and alleviation of suffering also represents a cornerstone within the ethos of nursing (Lindholm & Morse, 1993; Morse & Penrod, 1999; Rehnsfeldt & Eriksson, 2004). Rehnsfeldt and Eriksson (2004) stress that nurses need to urge an existential caring encounter with patients in order to alleviate suffering by making it bearable. In our findings, this is expressed through the experience of meaning in a joint togetherness. Likewise, Nygren Zotterman et al. (2016) stress that togetherness, in terms of attentiveness from nurses or other healthcare professionals, hence respect for “the other”, is the foundation of a caring relationship. Our findings add to this understanding by illustrating that sensitivity and an empathic understanding and imagination are pivotal in the care alliance in order to maintaining patients’ self and the experience of connectedness. With reference to the Danish philosopher K.E. Logstup and the Norwegian nursing philosopher K. Martinsen, Delmar (2008) stresses that in order to be a good and wise clinician, nursing practice starts with an existential insight where openness and an understanding of being morally obliged to risk and to go beyond oneself in the caring encounter with the patient needs to be present (Delmar, 2008). The present study illustrated the difficulty and complexity of being open and going beyond oneself in the practice of acute care is challenging. Nevertheless, introducing a lifeworld-sensitive approach within the already existing organization of care may enable Delmar’s (2008) request of risking and going beyond oneself in the caring practice.

### Limitations

The present paper presents a value framework for the humanization of care consisting of eight dimensions of lifeworld-led care that provide a sensitive direction for caring practice. The strength of the lifeworld-led care value framework is the explicit foundation in phenomenological philosophy. However, working with the eight dimensions is at times challenging, as they are analytical constructs. On one hand, they structured the analysis and provided us with the opportunity to ask new questions regarding the original material (Hammersley, 2010; Heaton, 2008). On the other hand, the dimensions were interrelated and thus at times difficult to separate from each other. Furthermore, the use of this specific theoretical value framework also created a certain frame for the questions asked during the analysis. Hence, other theoretical frameworks might have led to different interpretations.

As the data in this study were originally not collected for the purpose of a secondary analysis, it is important to acknowledge that when pre-collected data are reused, reflections upon “fit” and “context” are necessary (Hammersley, 2010). However, in this study ’fit’ and “context” were not issues that perturbed the analysis because all authors were very familiar with the datasets, and the dimensions of humanizing and dehumanizing care were “concepts” already debated within the author group during the work of the two previous studies.

## Conclusion

The present study illustrates aspects of how care to patients suffering from AUD admitted in AMUs can be both humanizing and dehumanizing. Our study shows that when nurses are successful in emphasizing the patients’ perspectives and understanding of their lived lives, the patients feel (more) humanly met. Thus, our findings underline that by letting patients’ perspective of well-being be the centre of care, an experience of mutual meaningfulness and sincerity within the provided care is nurtured. Consequently, lifeworld-led care has the potential to guide and enhance care to this group of vulnerable patients, also in the context of an AMU. Our study highlights that in order to provide a responsible and meaningful care, a profound and nuanced knowledge and understanding of the existential dimension faced by these patients is pivotal because nuances of the dehumanizing perspectives in this context are understood as humanizing, e.g., letting the patient be passive, hence acknowledging that passivity can be the way the individual patient participates. Thus, our study demonstrates the complexity of caring for patients suffering from AUD.

In conclusion, emphasizing the humanizing dimensions in acute nursing care for AUD patients as well as recognizing these patients’ lifeworlds are significant aspects to consider to create a substantial and meaningful caring focus for both nurses and patients.

## Implications to clinical practice

- Caring for patients suffering from AUD involve a distinct knowledge and attentiveness towards the patient’s lifeworld.
- Letting lifeworld-led care support the nurses in AMUs in addressing and meeting both challenges and care needs may enhance care to patients suffering from AUD.
- Letting patients’ perspective of well-being be the centre of care may nurture a mutual experience of meaningfulness and sincerity within the care alliance.
